# The impact of obesity and obstructive sleep apnea on vascular hemodynamics in men under 40: a 15-year retrospective cohort study

**DOI:** 10.1093/sexmed/qfag086

**Published:** 2026-09-14

**Authors:** Soarawee Weerasopone, Peyton J Coady, Arnaav Walia, Beatriz S Hernandez, Mohit Khera

**Affiliations:** Scott Department of Urology, Baylor College of Medicine, Houston, TX 77030, United States; Department of Urology, Bangkok Hospital, Bangkok 10310, Thailand; Scott Department of Urology, Baylor College of Medicine, Houston, TX 77030, United States; Scott Department of Urology, Baylor College of Medicine, Houston, TX 77030, United States; Scott Department of Urology, Baylor College of Medicine, Houston, TX 77030, United States; Scott Department of Urology, Baylor College of Medicine, Houston, TX 77030, United States

**Keywords:** erectile dysfunction, penile duplex ultrasound, ultrasonography, Doppler, duplex, arterial insufficiency, arterial occlusive diseases, obstructive sleep apnea, obesity, endothelial dysfunction, cardiovascular diseases, cardiovascular risk

## Abstract

**Introduction:**

Erectile dysfunction (ED) in men under 40 is frequently attributed to psychogenic causes, yet the contribution of objectively measured vascular disease in this age group is poorly characterized. We asked: in men under 40 undergoing penile duplex ultrasound (PDU) for ED, are obesity and obstructive sleep apnea (OSA) associated with arterial insufficiency (AI) independently of psychological factors, and do routine laboratory markers identify men with AI?

**Methods:**

Single-center retrospective cohort of men under 40 undergoing PDU between January 2010 and January 2025 (IRB H-51556, consent waived). Of 139 men, 33 were excluded for non-ED indications or neurogenic/anatomic confounders, leaving 106. Intracavernosal Trimix (papaverine 30 mg/phentolamine 1 mg/alprostadil 10 mcg per mL) was administered by the attending urologist; a single redose was permitted for absent response (*n* = 4, 3.8%), of whom only 1 subsequently reached an adequate response. Peak systolic velocity (PSV) and end-diastolic velocity (EDV) were recorded bilaterally at 5 and 15 minutes. Erectile response was recorded on a patient-reported 0-10 hardness scale; the validated four-point Erection Hardness Score was not administered. AI was classified side-specifically (PSV <30 cm/s). Venous leak (VL) was defined as EDV >5 cm/s at the timepoint of best PSV and analyzed as a binary variable, restricted to men with a final hardness score ≥ 5/10 (*n* = 79). OSA was ascertained by polysomnography (*n* = 5), home sleep testing (*n* = 2), or documented clinical diagnosis (*n* = 6); smoking, hypertension, diabetes, dyslipidemia, active stress, and psychiatric history were abstracted from the record.

**Results:**

Median age was 30.8 years; median BMI 25.7 kg/m^2^ (16.0% obese). AI was present in 18 (17.0%): 13 unilateral, 5 bilateral. VL was present in 31 of 79 (39.2%). AI was associated with BMI category (*P* = .016) and OSA (38.5% vs 14.0%, *P* = .036); OSA was associated with obesity (*P* = .001). In multivariable regression, OSA was associated with AI independently of BMI (OR 6.39, 95% CI 1.19–34.50, *P* = .031), although this interval is wide and rests on 13 OSA-positive men; BMI was not associated (*P* = .957). Neither active stress (*P* = .701) nor psychiatric history (*P* = .179) was associated with AI. Cholesterol, LDL, HbA1c, and testosterone were all non-discriminatory. VL was unrelated to BMI or OSA.

**Conclusions:**

Objectively measured arterial insufficiency affects a clinically meaningful minority of men under 40 with ED, is associated with OSA independently of BMI, and is not identified by routine laboratory testing or explained by psychological factors.

## Introduction

Erectile dysfunction (ED) affects 10%-16% of men under 40 years of age.^1^^,^^2^ Despite this prevalence, ED in young men is disproportionately attributed to psychogenic causes. The Diagnostic and Statistical Manual of Mental Disorders states that an age younger than 40 years is suggestive of a psychological etiology, a framing that may discourage objective vascular evaluation.^3^ Contemporary urologic guidance takes a different position: the American Urological Association directs that every man presenting with erectile dysfunction undergo a structured medical, sexual, and psychosocial history, physical examination, and selective laboratory testing, identifies erectile dysfunction as a marker of cardiovascular risk, and provides for specialized vascular testing where indicated, without exempting younger men from organic evaluation.^4^ Yet at least 15%-20% of young men with ED have an identifiable organic etiology.^5^

The pathophysiology of organic ED is predominantly vasculogenic, with endothelial dysfunction serving as the common link between erectile failure and systemic vascular disease.^6^ The artery size hypothesis posits that because penile cavernosal arteries (1-2 mm) are smaller than coronary arteries (3-4 mm), an equivalent degree of endothelial dysfunction produces clinically significant flow reduction in the penile vasculature before manifesting in larger arterial beds.^7^ Meta-analyses have demonstrated that ED independently predicts coronary heart disease (RR 1.50), stroke (RR 1.36), and all-cause mortality (RR 1.25).^8–10^ This predictive relationship is strongest in younger men.^11^^,^^12^

Obesity and obstructive sleep apnea (OSA) are interrelated metabolic conditions with established links to endothelial dysfunction. Obesity is associated with ED through insulin resistance, chronic inflammation, and reduced nitric oxide bioavailability, and studies using penile duplex ultrasound (PDU) have shown that body fat percentage independently predicts penile hemodynamic impairment.^13^^,^^14^ OSA independently causes endothelial dysfunction through intermittent hypoxia and oxidative stress.^15^ An individual patient data meta-analysis confirmed that severe OSA was independently associated with endothelial dysfunction (OR 2.27, 95% CI 1.12–4.60) after adjustment for obesity and other confounders.^16^ Approximately half of men with OSA have concurrent ED, suggesting shared pathophysiology.^17^

Despite these individual associations, no prior study has systematically evaluated the relationship between body mass index (BMI), OSA, and PDU-confirmed vascular pathology in a cohort composed exclusively of men under age 40. The unmet need is therefore an objective, hemodynamically anchored characterization of vascular ED in this age group, in which clinical practice most often defaults to a psychogenic presumption. This study was designed to answer the following questions. Among men under 40 undergoing standardized PDU for ED, are BMI and OSA associated with objectively measured arterial insufficiency, and does either retain an independent association after mutual adjustment and adjustment for documented psychological factors? Can standard laboratory markers—testosterone, HbA1c, and lipid parameters—discriminate men with arterial insufficiency from those without?

## Methods

### Study design, setting, and ethics

This single-center retrospective cohort study was conducted in an academic urology department. The institutional review board of the participating institution approved the study (Protocol H-51556) and granted a waiver of informed consent on the basis of the retrospective design and exclusive use of pre-existing clinical records. The study was conducted in accordance with the Declaration of Helsinki and is reported in accordance with the STROBE statement; a completed STROBE checklist accompanies this submission.^18^

### Participants

**Figure 1:**
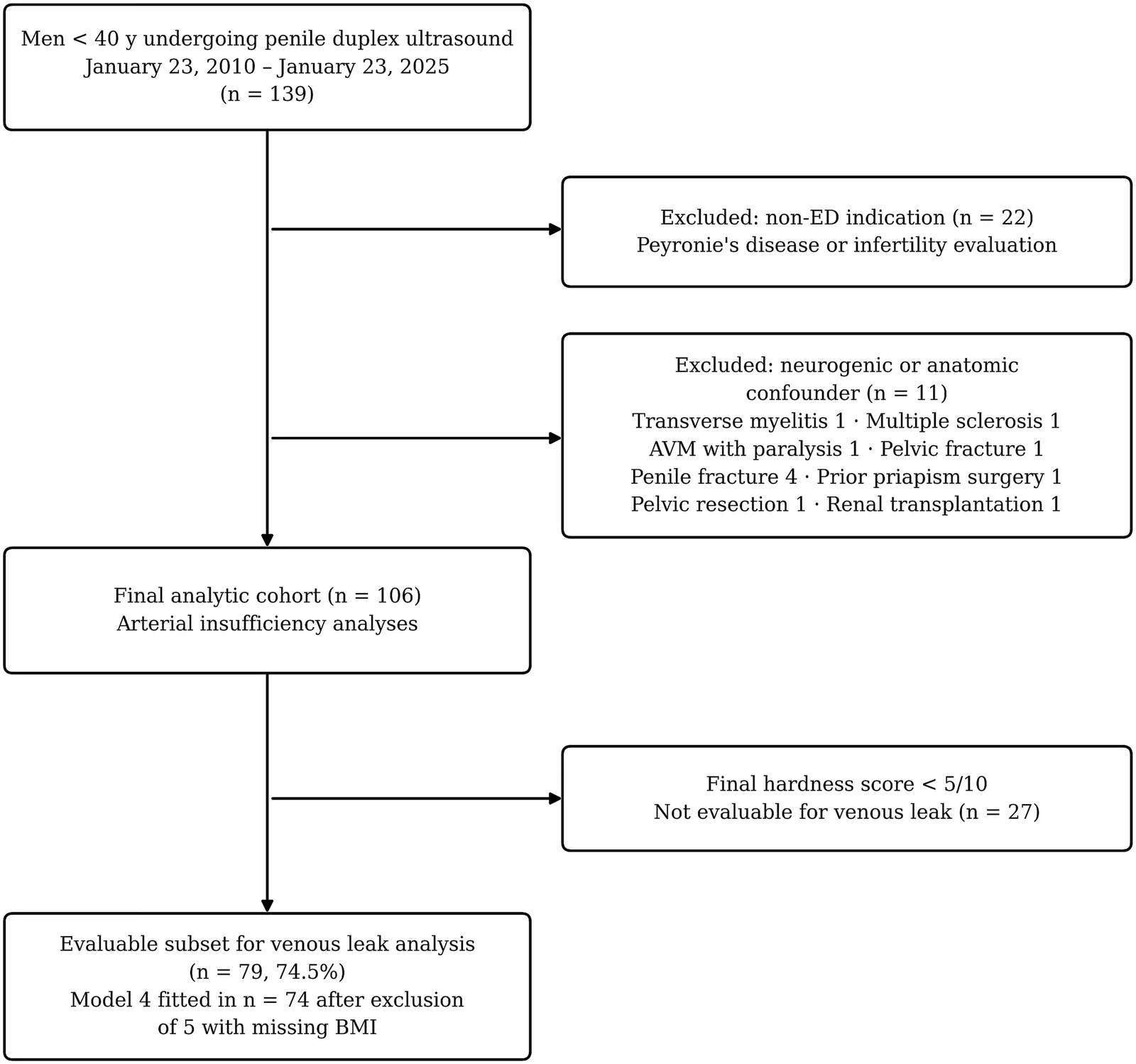
Patient flow diagram. Of 139 men under age 40 who underwent penile duplex ultrasound between January 23, 2010 and January 23, 2025, 22 were excluded for non-erectile-dysfunction indications and 11 for neurogenic or anatomic confounders, yielding a final cohort of 106. The evaluable subset for venous leak analysis comprised the 79 men (74.5%) achieving a final hardness score ≥ 5/10.

Medical records were reviewed for all male patients who underwent PDU between January 23, 2010 and January 23, 2025. Of 139 men under age 40, 22 were excluded for non-ED indications (Peyronie's disease or infertility evaluation) and 11 for neurogenic or anatomic confounders: transverse myelitis (*n* = 1), multiple sclerosis (*n* = 1), arteriovenous malformation with paralysis (*n* = 1), pelvic fracture (*n* = 1), penile fracture (*n* = 4), prior priapism requiring surgery (*n* = 1), pelvic resection (*n* = 1), and renal transplantation (*n* = 1, excluded because of potential iliac artery hemodynamic alteration from the transplant anastomosis). The final cohort comprised 106 patients (Figure 1).

### Penile duplex ultrasound protocol

All examinations followed a fixed institutional protocol. The attending urologist administered a single intracavernosal injection of Trimix (Empower Pharmacy, Houston, TX, USA), reconstituted at papaverine 30 mg/phentolamine 1 mg/alprostadil 10 mcg per mL, obtained from a single compounding pharmacy throughout the study period. Trimix was the sole vasoactive agent used in all 106 patients. Dose was selected by the attending urologist and was deliberately conservative given patient age, to reduce the risk of prolonged erection.

Following injection, the patient walked to one of two ultrasound suites, assigned by room availability, and rested for 5 minutes without stimulation of any kind. Scanning was performed by one of two dedicated ultrasound technicians active throughout the 15-year period, using an Aloka Arietta 60 (Room 1) or Aloka Arietta 65 (Room 2) system with a linear transducer operating at 5-15 MHz (ALOKA, as designated on the device label plate; the Arietta platform is currently distributed by Fujifilm Healthcare, Tokyo, Japan). Patients were examined supine. If the erectile response at 5 minutes was judged adequate, scanning proceeded immediately. If not, the patient was offered an additional 10 minutes of private self-stimulation specifically to avoid the need for redosing. If no response occurred thereafter, a single redose was permitted; no patient received more than two injections. Patients remained in the clinic area after imaging. A rigid erection persisting approximately 1 hour after injection without interval detumescence prompted on-site pharmacologic reversal. This timing reflected consistent departmental practice rather than a written protocol or a validated threshold; it was adopted because the risk of hypoxic cavernosal injury rises with the duration of an unrelieved rigid erection, because aspiration and pharmacologic reversal become progressively more difficult with time, and because patients had completed imaging and could not practicably be observed in clinic for the 4-hour interval that defines ischemic priapism. Reversal used phenylephrine, supplied at 10 mg/mL and diluted in 0.9% sodium chloride to a working concentration of 1 mg/mL; 500 μg (0.5 mL) was injected intracavernosally every 5 minutes until detumescence, with one or two doses sufficient in most instances and three or four required occasionally, under monitoring for hypertension and reflex bradycardia. These events represent pharmacologically induced prolonged erections, which current guidance defines as lasting less than 4 hours and distinguishes explicitly from ischemic priapism, for which separate management protocols apply; the frequency reported here is therefore not a priapism rate and is not comparable with published priapism or test-dosing reversal series.^19^

Doppler measurements of peak systolic velocity (PSV) and end-diastolic velocity (EDV) were obtained from both cavernosal arteries at 5 and 15 minutes post-injection. For each side, the timepoint yielding the highest PSV was selected, and the EDV recorded at that same timepoint was used, preserving the physiological relationship between the two measurements.^20^^,^^21^

### Erectile response and hemodynamic definitions

Erectile response was recorded as a patient-reported hardness score on a 0-10 scale. The validated four-point Erection Hardness Score instrument was not administered. All analytic decisions used the raw 0-10 score, and the evaluable threshold was a final score ≥ 5/10 (post-redose value where applicable), yielding 79 patients (74.5%).

Arterial insufficiency (AI) was classified side-specifically from raw PSV data: normal (bilateral PSV ≥30 cm/s), unilateral AI (one corpus cavernosum <30 cm/s with the contralateral ≥30 cm/s), or bilateral AI (both <30 cm/s). Venous leak (VL) was defined as EDV >5 cm/s at the timepoint of best PSV in either corpus cavernosum and was analyzed as a binary variable, restricted to the 79 men achieving a final hardness score ≥ 5/10 to avoid false-positive diagnoses from incomplete smooth muscle relaxation.

### Variables

Clinical variables extracted by structured chart review included age, BMI, race and ethnicity, ED duration, phosphodiesterase type 5 inhibitor response, smoking status, hypertension, diabetes, dyslipidemia, OSA, active psychosocial stress, psychiatric history, and hormonal therapy. BMI was categorized as normal weight (<25.0), overweight (25.0-29.9), or obese (≥30.0 kg/m^2^). Comorbidities other than OSA were ascertained from documented clinical diagnoses in the medical record. OSA was ascertained by polysomnography (*n* = 5), home sleep apnea testing (*n* = 2), or documented clinical diagnosis without confirmatory testing (*n* = 6); apnea–hypopnea index severity and treatment adherence data were not available. Active stress and psychiatric history were documented by the attending urologist at the index encounter. Laboratory values comprised total testosterone, free testosterone, HbA1c, and a lipid panel, all obtained as part of routine clinical care without protocolized timing or fasting requirements.

### Statistical analysis

Continuous variables were non-normally distributed (Shapiro–Wilk *P* < .05) and are reported as median (interquartile range); categorical variables are reported as n (%). Categorical associations were tested with Fisher's exact test with Cramér’s V effect sizes. Continuous comparisons used Kruskal–Wallis and Mann–Whitney U tests, and Spearman's rank correlation assessed continuous associations. For comparisons involving the smallest groups, asymptotic p-values were verified against Monte Carlo estimates based on 10 000 sampled tables with 99% confidence intervals; all asymptotic and Monte Carlo results were concordant. Binary logistic regression evaluated predictors of AI in sequential models: Model 1 (BMI, continuous and categorical specifications), Model 2 (BMI + OSA), Model 3 (BMI + OSA + active stress + psychiatric history), and Model 3a (sensitivity, excluding psychiatric history). Model 4 evaluated BMI + OSA as predictors of VL. Model fit was assessed by omnibus tests, Nagelkerke *R*^2^ and Hosmer–Lemeshow tests. Receiver operating characteristic analysis compared BMI with laboratory markers. A sensitivity analysis excluded the 7 patients on hormonal therapy. Analyses used IBM SPSS Statistics version 29.0 (IBM Corp., Armonk, NY, USA); two-sided *P* < .05 was considered significant.

## Results

### Cohort characteristics

Median age was 30.8 years (IQR 25.5–35.3) and median ED duration 2.4 years (IQR 1.0–5.4). The cohort was White 46.2%, Black 12.3%, Asian 3.8%, other 25.5%, declined 12.3%, and 75.5% non-Hispanic/Latino; 66.0% were single. Median BMI was 25.7 kg/m^2^ (IQR 22.6–28.8; *N* = 100), with 42.0% normal weight, 42.0% overweight, and 16.0% obese. Comorbidities included hypertension (13.2%), diabetes (6.6%), dyslipidemia (13.2%), OSA (12.3%), active smoking (13.2%), active stress (13.2%), and any psychiatric history (36.8%). Seven patients (6.6%) were on hormonal therapy. Full baseline characteristics and laboratory values appear in Table 1.

**Table 1:** Baseline demographics and clinical characteristics (N = 106).

**Variable**	**Value**
**Age at penile duplex ultrasound (years)**	
Median (IQR)	30.8 (25.5–35.3)
Range	16.7–39.5
**ED duration (years), N = 106**	
Median (IQR)	2.4 (1.0–5.4)
Range	0–17.1
**Race, n (%)**	
White	49 (46.2%)
Black	13 (12.3%)
Asian	4 (3.8%)
Other	27 (25.5%)
Declined	13 (12.3%)
**Ethnicity, n (%)**	
Non-Hispanic/Latino	80 (75.5%)
Hispanic/Latino	17 (16.0%)
Unknown	9 (8.5%)
**Relationship status, n (%)**	
Single	70 (66.0%)
Married	23 (21.7%)
Divorced	1 (0.9%)
Significant other	1 (0.9%)
Unknown	11 (10.4%)
**BMI (kg/m** ^**2**^ **), N = 100**	
Median (IQR)	25.7 (22.6–28.8)
Range	18.0–41.8
Normal weight (< 25.0)	42 (42.0%)
Overweight (25.0–29.9)	42 (42.0%)
Obese (≥ 30.0)	16 (16.0%)
**Comorbidities, n (%)**	
Hypertension	14 (13.2%)
Diabetes mellitus	7 (6.6%)
Dyslipidemia	14 (13.2%)
Obstructive sleep apnea (any)	13 (12.3%)
— Polysomnography-confirmed	5
— Home sleep apnea test–confirmed	2
— Documented clinical diagnosis, no confirmatory testing	6
**Smoking status, n (%)**	
Never	82 (77.4%)
Active	14 (13.2%)
Former	10 (9.4%)
**Psychiatric history, n (%)**	
None	67 (63.2%)
Depression	23 (21.7%)
Anxiety	11 (10.4%)
ADHD	3 (2.8%)
Bipolar disorder	2 (1.9%)
**Active psychosocial stress, n (%)**	14 (13.2%)
**Hormonal therapy (N = 106), n (%)**	
Any hormonal therapy	7 (6.6%)
— Testosterone injection	1
— Testosterone topical	3
— Clomiphene	3
**PDE5 inhibitor response, n (%)**	
Full response	3 (2.8%)
Partial response	55 (51.9%)
No response	5 (4.7%)
Never tried	43 (40.6%)
**Laboratory values, median (IQR)**	
Total testosterone (ng/dL), N = 99	441 (354–579)
Free testosterone (ng/dL), N = 91	8.4 (7.0–11.2)
HbA1c (%), N = 47	5.4 (5.0–5.7)
Total cholesterol (mg/dL), N = 49	184.0 (158.0–207.5)
LDL cholesterol (mg/dL), N = 48	105.5 (81.3–128.8)
HDL cholesterol (mg/dL), N = 49	48.0 (41.3–58.5)
Triglycerides (mg/dL), N = 49	125.0 (73.5–175.5)

Abbreviations: ED = erectile dysfunction; BMI = body mass index; PDE5 = phosphodiesterase type 5.

Data are median (interquartile range) for continuous variables and n (%) for categorical variables. All continuous variables were non-normally distributed (Shapiro–Wilk *P* < .05). BMI was unavailable for 6 patients. Comorbidities other than obstructive sleep apnea were ascertained from documented clinical diagnoses in the medical record. Laboratory samples were obtained during routine clinical care without protocolized timing or documented fasting status.

### Penile duplex ultrasound findings

Median Trimix volume was 0.05 mL (IQR 0.05–0.05; range 0.02-0.25), corresponding to a median of papaverine 1.5 mg, phentolamine 0.05 mg, and alprostadil 0.5 mcg (range 0.6-7.5 mg, 0.02-0.25 mg, and 0.2-2.5 mcg, respectively). Four patients (3.8%) required a single redose; all had an initial hardness score of 0, redose was administered 5-32 minutes later at 0.05-0.1 mL, and 3 of the 4 remained below the evaluable threshold and were excluded from VL analysis. Twelve patients (11.3%) underwent pharmacologic reversal of a prolonged erection at approximately 1 hour, none of whom were among the redosed patients, and no pain events were recorded.

Median best PSV was 46.3 cm/s (right) and 44.6 cm/s (left). AI was identified in 18 patients (17.0%): 13 (12.3%) unilateral and 5 (4.7%) bilateral. Among the 79 evaluable men, VL was present in 31 (39.2%). Hemodynamic findings are summarized in Table 2.

**Table 2:** Penile duplex ultrasound protocol parameters and hemodynamic findings (N = 106).

**Variable**	**Value**
**Trimix dose—volume (mL)**	
Median (IQR)	0.05 (0.05–0.05)
Mean ± SD	0.063 ± 0.041
Range	0.02–0.25
**Trimix dose—absolute drug mass**	
Papaverine (mg), median (range)	1.5 (0.6–7.5)
Phentolamine (mg), median (range)	0.05 (0.02–0.25)
Alprostadil (mcg), median (range)	0.5 (0.2–2.5)
**Protocol events, n (%)**	
Single redose required	4 (3.8%)
— Remaining below evaluable threshold after redose	3 of 4
Pharmacologic reversal of prolonged erection at ~1 hour	12 (11.3%)
Pain events recorded	0 (0%)
**Final patient-reported hardness score (0–10)**	
Median (IQR), N = 106	6.0 (4.0–8.0)
Final score ≥ 5/10 (evaluable subset), n (%)	79 (74.5%)
**Peak systolic velocity (cm/s), median (IQR), N = 106**	
Right corpus cavernosum	46.3 (35.1–64.2)
Left corpus cavernosum	44.6 (35.6–60.8)
**End-diastolic velocity at best PSV (cm/s), median (IQR), evaluable subset N = 79**	
Right corpus cavernosum	0.2 (−8.6 to 7.6)
Left corpus cavernosum	0.0 (−7.9 to 5.0)
**Arterial insufficiency classification, n (%), N = 106**	
Normal (bilateral PSV ≥ 30 cm/s)	88 (83.0%)
Unilateral arterial insufficiency	13 (12.3%)
Bilateral arterial insufficiency	5 (4.7%)
Any arterial insufficiency	18 (17.0%)
**Venous leak, n (%), evaluable subset N = 79**	
No venous leak	48 (60.8%)
Venous leak present	31 (39.2%)

Abbreviation: PSV = peak systolic velocity. Trimix (Empower Pharmacy, Houston, TX, USA) was reconstituted at papaverine 30 mg/phentolamine 1 mg/alprostadil 10 mcg per mL and was the sole vasoactive agent in all 106 patients. For each corpus cavernosum, the timepoint (5 or 15 minutes post-injection) yielding the highest PSV was selected, and the end-diastolic velocity recorded at that same timepoint was used. Erectile response was recorded on a patient-reported 0–10 hardness scale; the validated four-point Erection Hardness Score instrument was not administered. The evaluable subset comprised the 79 men with a final hardness score ≥ 5/10 (post-redose value where applicable). Venous leak was defined as end-diastolic velocity > 5 cm/s at the timepoint of best PSV in either corpus cavernosum and is reported as a binary variable, consistent with veno-occlusive dysfunction being a global corporal rather than a vessel-specific process. Prolonged erection was managed with intracavernosal phenylephrine, diluted from 10 mg/mL to a working concentration of 1 mg/mL in 0.9% sodium chloride and given as 500 μg (0.5 mL) every 5 minutes until detumescence, with monitoring for hypertension and reflex bradycardia. None of the 12 patients were among the 4 redosed patients, so the interval was measured from a single injection. The approximately 1-hour timing reflected consistent departmental practice rather than a written protocol or validated threshold; these were pharmacologically induced prolonged erections, defined as lasting less than 4 hours and distinct from ischemic priapism, and this frequency is therefore not a priapism rate.

### Obesity, OSA, and arterial insufficiency

BMI category was associated with AI (Fisher's *P* = .016, Cramér's V = 0.286): bilateral AI occurred in 25.0% of obese men versus 2.4% of normal-weight and 0% of overweight men (Table 3, Supplementary Figure S1). BMI was inversely correlated with right PSV (*ρ* = −0.206, *P* = .040), and right PSV across BMI categories was borderline (H = 5.438, *P* = .066; Monte Carlo *P* = .063, 99% CI 0.056–0.069), with medians of 54.8, 45.7, and 41.9 cm/s across normal-weight, overweight, and obese groups.

**Table 3:** Association between BMI category and arterial insufficiency (N = 100).

**Arterial insufficiency**	**Normal weight (*n* = 42)**	**Overweight (*n* = 42)**	**Obese (*n* = 16)**	***P*-value**
Normal	36 (85.7%)	37 (88.1%)	11 (68.8%)	**.016**
Unilateral AI	5 (11.9%)	5 (11.9%)	1 (6.3%)	
Bilateral AI	1 (2.4%)	0 (0.0%)	4 (25.0%)	

Abbreviation: AI = arterial insufficiency. Fisher's exact test; Cramér's V = 0.286. Six patients were excluded for missing BMI data. Bolded *P*-value indicates statistical significance.

OSA was associated with AI (Fisher's *P* = .036, Cramér's V = 0.234): 38.5% of men with OSA had AI versus 14.0% without (Table 4). OSA was strongly associated with obesity, present in 43.8% of obese men versus 9.5% of overweight and 4.8% of normal-weight men (Fisher's exact *P* = .001, Cramér's V = 0.404) (Supplementary Table S1, Supplementary Figure S2). Left PSV was lower in men with OSA (median 38.1 versus 47.7 cm/s; Monte Carlo *P* = .026, 99% CI 0.022–0.030), while right PSV did not differ (*P* = .188).

**Table 4:** Association between obstructive sleep apnea and arterial insufficiency (N = 106).

**Arterial insufficiency**	**No OSA (*n* = 93)**	**OSA (*n* = 13)**	***P*-value**
Normal	80 (86.0%)	8 (61.5%)	**.036**
Unilateral AI	10 (10.8%)	3 (23.1%)	
Bilateral AI	3 (3.2%)	2 (15.4%)	

Abbreviations: AI = arterial insufficiency; OSA = obstructive sleep apnea. Fisher's exact test; Cramér's V = 0.234. OSA was defined as any of polysomnography-confirmed (*n* = 5), home sleep apnea test–confirmed (*n* = 2), or documented clinical diagnosis without confirmatory testing (*n* = 6). Apnea–hypopnea index severity and treatment adherence data were unavailable. Bolded *P*-value indicates statistical significance.

### Psychological factors and laboratory markers

Neither active stress (*P* = .701) nor psychiatric history (*P* = .179) was associated with AI, and PSV did not differ by either factor (Supplementary Tables S2 and S3). Traditional cardiovascular diagnoses were not associated with AI: hypertension (*P* = .575), diabetes (*P* = .711), dyslipidemia (*P* = .309), and smoking (*P* = .854). Laboratory markers were likewise non-significant—total cholesterol (*P* = .830), LDL (*P* = .445), HbA1c (*P* = .243), and triglycerides (*P* = .085)—with the exception of low HDL (*P* = .035, Cramér's V = 0.478), an association limited by the small subgroup with available measurements (Supplementary Tables S4 and S5). Age, Trimix volume, and final hardness score did not differ across AI categories (Supplementary Table S6).

Receiver operating characteristic analysis showed BMI (AUC 0.584, 95% CI 0.42–0.75) outperforming free testosterone (0.563), HbA1c (0.557), total testosterone (0.510), and LDL (0.344), though none reached significance; the optimal BMI cutoff was 29.4 kg/m^2^ (Youden index 0.259) (Supplementary Figure S3).

### Multivariable analysis

**Figure 2:**
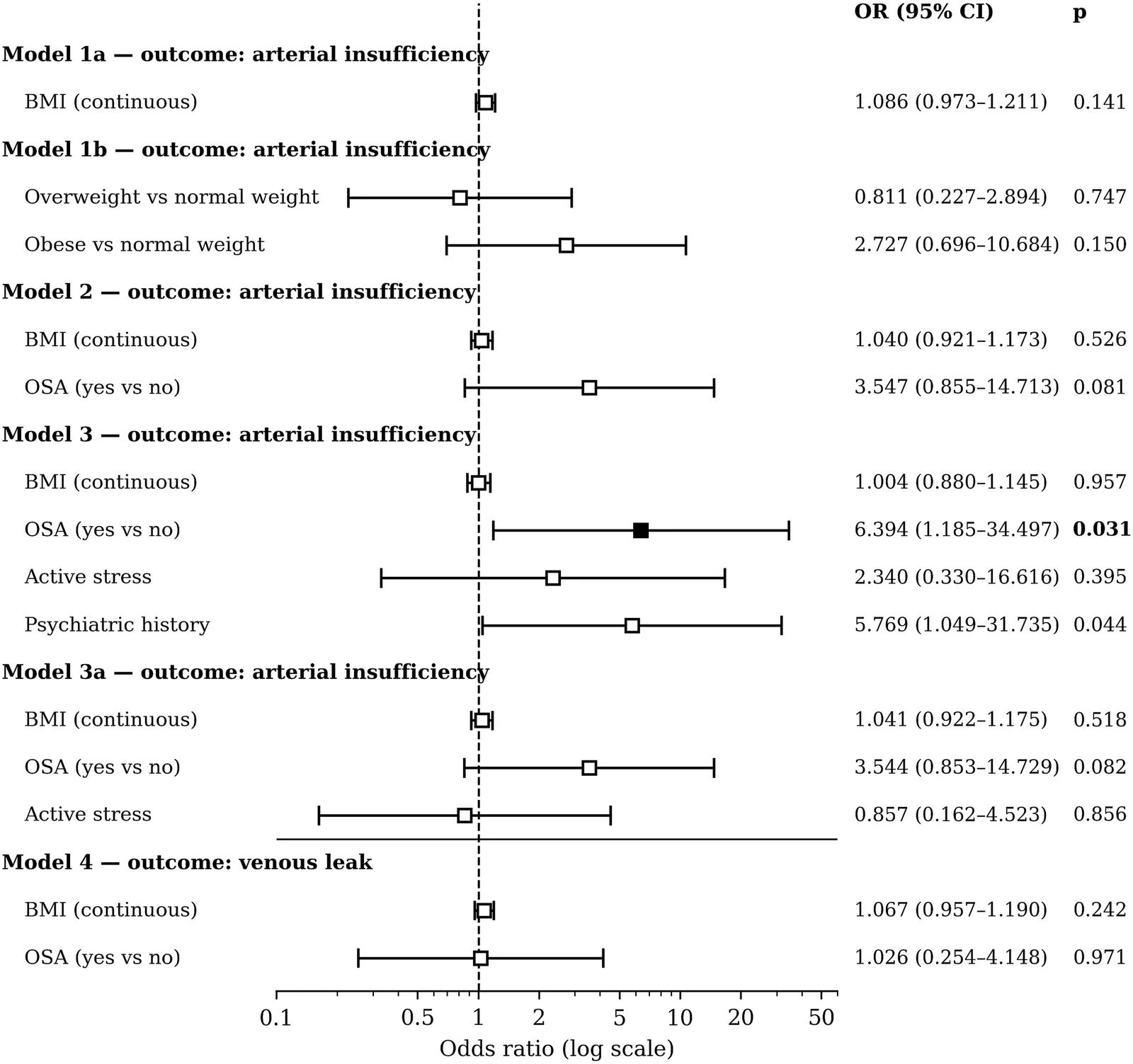
Forest plot of odds ratios with 95% confidence intervals from the sequential binary logistic regression models predicting arterial insufficiency (Models 1, 2, 3, and 3a) and venous leak (Model 4). The vertical reference line marks an odds ratio of 1.0. Obstructive sleep apnea was the strongest significant independent predictor of arterial insufficiency (Model 3: OR 6.394, 95% CI 1.185–34.497, *P* = .031).

BMI alone did not predict AI (Model 1 continuous: OR 1.086, *P* = .141; obese versus normal weight: OR 2.727, *P* = .150). Adding OSA attenuated BMI (OR 1.040, *P* = .526) while OSA approached significance (OR 3.547, *P* = .081). In Model 3 the overall model was significant (omnibus *P* = .037, Nagelkerke *R*^2^ = 0.166), with OSA the strongest independent predictor (OR 6.394, 95% CI 1.185–34.497, *P* = .031), BMI non-significant (*P* = .957), and active stress non-significant (*P* = .395). This estimate derives from only 13 OSA-positive men, of whom 6 had no confirmatory sleep testing; the width of the confidence interval indicates low precision, and a single discordant outcome would materially change the estimate. Psychiatric history reached nominal significance (OR 5.769, *P* = .044) but was driven by sparse cells and unsupported by bivariate testing; in Model 3a the model lost significance (omnibus *P* = .172) and OSA again showed a trend (*P* = .082), indicating that psychiatric history functioned as a statistical adjustor rather than an independent predictor. Full model output appears in Table 5 and is displayed in Figure 2.

**Table 5:** Sequential binary logistic regression models predicting arterial insufficiency and venous leak.

**Predictor**	**B**	**Wald**	***P*-value**	**OR**	**95% CI**
**Model 1a: BMI (continuous) alone—outcome AI, N = 100**					
BMI (continuous)	0.082	2.170	0.141	1.086	0.973–1.211
**Model 1b: BMI (categorical)—outcome AI, N = 100**					
Overweight vs normal weight	−0.210	0.104	0.747	0.811	0.227–2.894
Obese vs normal weight	1.003	2.074	0.150	2.727	0.696–10.684
**Model 2: BMI + OSA—outcome AI, N = 100**					
BMI (continuous)	0.039	0.401	0.526	1.040	0.921–1.173
OSA (yes vs no)	1.266	3.042	0.081	3.547	0.855–14.713
**Model 3: BMI + OSA + active stress + psychiatric history—outcome AI, N = 100**					
BMI (continuous)	0.004	0.003	0.957	1.004	0.880–1.145
OSA (yes vs no)	1.855	4.654	**0.031**	**6.394**	**1.185–34.497**
Active stress	0.850	0.722	0.395	2.340	0.330–16.616
Psychiatric history	1.752	4.059	0.044	5.769	1.049–31.735
**Model 3a (sensitivity, psychiatric history removed)—outcome AI, N = 100**					
BMI (continuous)	0.040	0.417	0.518	1.041	0.922–1.175
OSA (yes vs no)	1.265	3.029	0.082	3.544	0.853–14.729
Active stress	−0.155	0.033	0.856	0.857	0.162–4.523
**Model 4: BMI + OSA—outcome venous leak, evaluable subset N = 74**					
BMI (continuous)	0.065	1.370	0.242	1.067	0.957–1.190
OSA (yes vs no)	0.026	0.001	0.971	1.026	0.254–4.148
**Model fit statistics**					
**Model**	**Omnibus χ** ^**2**^	**Omnibus *P***	**Nagelkerke *R*** ^**2**^	**Hosmer–Lemeshow *P***	
Model 3 (AI)	10.19	**.037**	0.166	> .74	
Model 3a (AI)	—	.172	—	> .74	
Model 4 (venous leak)	1.783	.410	0.032	.165	

Abbreviations: AI = arterial insufficiency; OSA = obstructive sleep apnea; OR = odds ratio; CI = confidence interval. Reference category for categorical BMI is normal weight. Model 4 was fitted in 74 of the 79 evaluable patients; 5 were excluded for missing BMI. Bolded values indicate statistical significance (*P* < .05). The nominal significance of psychiatric history in Model 3 was driven by sparse cells and was not supported on bivariate testing (Fisher's exact *P* = .179); its removal in Model 3a rendered the overall model non-significant.

### Venous leak

VL was not associated with BMI category (*P* = .162, V = 0.217), OSA (*P* = .199, V = 0.215), active stress (*P* = .899, V = 0.059), or psychiatric history (*P* = .492, V = 0.132). Continuous EDV at best PSV likewise did not differ by AI status (right *P* = .388; left *P* = .196), OSA status (right Monte Carlo *P* = .292; left *P* = .716), active stress (right *P* = .846; left *P* = .458), or BMI category (right Monte Carlo *P* = .352; left *P* = .855). Model 4 was non-significant overall (omnibus χ^2^ = 1.783, *P* = .410; Nagelkerke *R*^2^ = 0.032; Hosmer–Lemeshow *P* = .165), with neither BMI (OR 1.067, 95% CI 0.957–1.190, *P* = .242) nor OSA (OR 1.026, 95% CI 0.254–4.148, *P* = .971) predictive.

### Sensitivity analysis

Excluding the 7 patients on hormonal therapy did not alter the principal findings (AI × BMI: Fisher’s *P* = .015; VL × BMI: *P* = .244) (Supplementary Table S7).

## Discussion

This 15-year study of men under 40 undergoing standardized PDU yields three principal findings: objectively confirmed AI was present in 17.0%; obesity and OSA formed a metabolic cluster associated with AI, with OSA the strongest significant independent predictor; and standard laboratory markers failed to identify this vascular disease.

The 17.0% prevalence of PDU-confirmed AI does not by itself refute the view that ED in young men is frequently psychogenic, and the present data should not be read that way. It does establish that a substantial minority of young men referred for evaluation harbor demonstrable arterial disease, a proportion consistent with prior estimates of organic etiology in 15%-45% of young men with ED.^22^ The side-specific AI classification may capture a transitional stage of vascular disease obscured by conventional binary reporting, consistent with recommendations to interpret raw hemodynamic data from each corpus cavernosum independently.^20^^,^^21^

The central finding is that OSA emerged as the strongest significant independent predictor of AI while BMI lost predictive value once OSA entered the model—a pattern consistent with OSA mediating the BMI–AI relationship. This was not a formal mediation analysis with bootstrapped indirect effects, and the wide confidence interval reflects modest sample size, so the pattern is hypothesis-generating. It is nonetheless biologically plausible: obesity downregulates vascular PPAR-γ, and combined with chronic intermittent hypoxia produces exaggerated oxidative stress and endothelial dysfunction.^23^ An individual patient data meta-analysis confirmed that severe OSA is independently associated with endothelial dysfunction after adjustment for obesity,^16^ and the American Heart Association recognizes central adiposity as the link between OSA and metabolic syndrome through shared inflammatory and endothelial mechanisms.^24^ Clinically, BMI functions as an accessible marker flagging patients at risk for OSA-mediated vascular injury, with the optimal cutoff of 29.4 kg/m^2^ closely approximating the standard obesity threshold.

Venous leak, by contrast, was unrelated to BMI, OSA, or any metabolic predictor across categorical, continuous, and multivariable specifications. This null result is consistent with the prevailing understanding of veno-occlusive dysfunction as a global corporal process arising from smooth muscle and tunical structural change rather than from the metabolic milieu. That conceptualization also explains why venous leak is reported here as a binary finding: because the underlying mechanism is corporal rather than vessel-specific, a side-specific classification analogous to that used for arterial insufficiency is not physiologically defensible, and the initially recorded unilateral and bilateral categories were collapsed accordingly.

The inability of standard laboratory markers to discriminate AI status is notable. Testosterone, HbA1c, and lipid parameters all failed, consistent with Yao et al.^25^ who found increased carotid intima-media thickness and reduced flow-mediated vasodilation in young men with ED despite normal laboratory values. This dissociation supports the concept that ED may represent the earliest clinical manifestation of endothelial dysfunction, preceding detectable metabolic derangement, in keeping with the artery size hypothesis.^7^ Neither active stress nor psychiatric history predicted AI on any analysis, which carries a practical implication: a young man reporting stress should not be presumed to have purely psychogenic ED, since organic and psychogenic etiologies frequently coexist.

These findings align with evidence positioning ED as an early marker of cardiovascular risk. In the Olmsted County cohort of men aged 40-79, ED was associated with incident coronary artery disease, with the association strongest among the youngest men studied—an age-modification finding rather than one confined to a single decade.^26^ The present study extends this paradigm to men in their twenties and thirties, identifying a phenotype—young, obese men with OSA and subclinical AI undetectable by routine laboratory testing—that may represent a window for early cardiovascular intervention through weight reduction and OSA treatment.^27^^,^^28^ A practical implication follows. Because OSA was the exposure most strongly associated with arterial insufficiency, and because 6 of the 13 affected men had never undergone confirmatory sleep testing, a validated screening instrument warrants incorporation into the evaluation of young men presenting with erectile dysfunction who are obese or otherwise at risk. The STOP-BANG questionnaire carries the highest pooled sensitivity among validated instruments, 90% for moderate and 93% for severe disease, with correspondingly low specificity of approximately 35%; this profile suits it to ruling out rather than diagnosing the condition, and therefore to selecting men for confirmatory testing.^29^

Several limitations apply. This single-center retrospective study is subject to referral bias, and the sample size limits multivariable power, as reflected in wide confidence intervals. OSA ascertainment was heterogeneous, with only 5 of 13 diagnoses polysomnographically confirmed, and neither apnea–hypopnea index severity nor treatment adherence was available; this is the most consequential limitation, since the principal finding rests on this exposure. Laboratory sampling was not protocolized: blood draws occurred across the working day rather than in the morning, so testosterone values cannot be interpreted as standardized morning measurements, and lipid and HbA1c samples were drawn at the same non-standardized times without documented fasting status. Laboratory ascertainment was also sparse: HbA1c was available in 47 of 106 patients (44.3%), a complete lipid panel in 48 (45.3%), total testosterone in 99 (93.4%), and free testosterone in 91 (85.8%), so these analyses are underpowered and exploratory. No correction for multiple comparisons was applied across the several bivariate and exploratory analyses, and nominal p-values should be interpreted accordingly; the isolated low-HDL association, resting on 49 patients, is most consistent with a chance finding. Erectile response was captured on a patient-reported 0-10 scale rather than the validated four-point instrument. Stimulation status varied by protocol design—patients with an inadequate response at 5 minutes underwent a 10-minute self-stimulation step—and the number of patients requiring this step was not documented in the medical record. The timing of pharmacologic reversal likewise reflected consistent but non-protocolized practice rather than a predefined or validated threshold, exact injection-to-intervention intervals were not systematically recorded, and the resulting frequency cannot be compared directly with published priapism or test-dosing reversal rates. There was no non-ED control group, and the cross-sectional hemodynamic assessment precludes causal inference. Finally, although the cohort was racially and ethnically diverse, the findings derive from a single U.S. academic referral center. Future work should pursue prospective enrollment with polysomnographic confirmation and severity grading, protocolized morning fasting laboratory sampling, formal causal mediation analysis, and longitudinal cardiovascular follow-up.

## Conclusion

Objectively confirmed arterial insufficiency was present in 17.0% of men under 40 evaluated for erectile dysfunction and was associated with obesity and obstructive sleep apnea rather than with documented psychological factors, with obstructive sleep apnea the strongest significant independent predictor.^16^^,^^23^^,^^24^ Venous leak was unrelated to the metabolic cluster across all specifications, consistent with a distinct global corporal mechanism. Standard laboratory markers failed to identify subclinical arterial disease, supporting erectile dysfunction as a potential early manifestation of endothelial dysfunction.^7^^,^^25^ Prospective studies with polysomnographic confirmation are needed to determine whether these abnormalities predict future cardiovascular events and whether weight reduction and sleep apnea treatment reverse early vascular impairment.^27^^,^^28^

## Supplementary Material

Supplementary-Figure-S1-AI-by-BMI-Category_qfag086

Supplementary-Figure-S2-OSA-by-BMI-Category_qfag086

Supplementary-Figure-S3-ROC-Curves_qfag086

Supplementary-Table-S1-Association-Between-Obstructive-Sleep-Apnea-and-BMI_qfag086

Table-S2-Association-Between-Psychological-Factors-and-Arter_qfag086

Supplementary-Table-S3-Non-Parametric-Comparisons-of-Hemodynamic-Parameter_qfag086

Supplementary-Table-S4-Median-Hemodynamic-Values-by-Exposure-Group_qfag086

Supplementary-Table-S5-Association-Between-Traditional-Risk-Factors-Labora_qfag086

Supplementary-Table-S6-Age-Trimix-Volume-and-Final-Hardness-Score-Across-A_qfag086

Supplementary-Table-S7-Sensitivity-Analysis-Excluding-Patients-on-Hormonal_qfag086

## Data Availability

The data underlying this article cannot be shared publicly because they contain protected health information. De-identified data may be made available by the corresponding author on reasonable request, subject to approval by the Institutional Review Board of Baylor College of Medicine.

## Notes

**Meeting Presentation:** Accepted for podium presentation at the 2026 Annual Meeting of the South Central Section of the American Urological Association, September 9–12, 2026, Tucson, Arizona, USA.
