# Supplementary material for: The impact of obesity and obstructive sleep apnea on vascular hemodynamics in men under 40: a 15-year retrospective cohort study
Source: Sex Med. 2026 Sep 14;14(6):qfag086. doi: 10.1093/sexmed/qfag086 (PMC13574288; doi:10.1093/sexmed/qfag086)
Supplement: Supplementary file 1 [file supplementary-table-s1-association-between-obstructive-sleep-apnea-and-bmi_qfag086.docx]

**Supplementary Table S1. Association Between Obstructive Sleep Apnea and BMI Category (N = 100)**

| **OSA status** | **Normal weight (n = 42)** | **Overweight (n = 42)** | **Obese (n = 16)** | **p-value** |
| --- | --- | --- | --- | --- |
| No OSA | 40 (95.2%) | 38 (90.5%) | 9 (56.3%) | **0.001** |
| OSA present | 2 (4.8%) | 4 (9.5%) | 7 (43.8%) |  |

*OSA = obstructive sleep apnea. Fisher's exact test; Cramér's V = 0.404. Six patients were excluded for missing BMI data.*
