# Supplementary material for: The impact of obesity and obstructive sleep apnea on vascular hemodynamics in men under 40: a 15-year retrospective cohort study
Source: Sex Med. 2026 Sep 14;14(6):qfag086. doi: 10.1093/sexmed/qfag086 (PMC13574288; doi:10.1093/sexmed/qfag086)
Supplement: Supplementary file 2 [file supplementary-table-s3-non-parametric-comparisons-of-hemodynamic-parameter_qfag086.docx]

**Supplementary Table S3. Non-Parametric Comparisons of Hemodynamic Parameters**

| **Comparison** | **Test statistic** | **Asymptotic p** | **Monte Carlo p (99% CI)** |
| --- | --- | --- | --- |
| **PSV across BMI categories (Kruskal–Wallis, N = 100)** |  |  |  |
| Right PSV | H = 5.438, df = 2 | 0.066 | 0.063 (0.056–0.069) |
| Left PSV | H = 1.979, df = 2 | 0.372 | 0.366 (0.353–0.378) |
| **PSV across AI categories (Kruskal–Wallis, N = 106)** |  |  |  |
| Right PSV | H = 38.072, df = 2 | < 0.001 | — |
| Left PSV | H = 21.202, df = 2 | < 0.001 | — |
| **PSV by OSA status (Mann–Whitney U, N = 106; 93 vs 13)** |  |  |  |
| Right PSV | U = 468, Z = −1.315 | 0.189 | 0.188 (0.178–0.198) |
| Left PSV | U = 378, Z = −2.182 | **0.029** | **0.026 (0.022–0.030)** |
| **PSV by active stress (Mann–Whitney U, N = 106)** |  |  |  |
| Right PSV | U = 484, Z = −1.493 | 0.135 | — |
| Left PSV | U = 623.5, Z = −0.191 | 0.848 | — |
| **PSV by psychiatric history (Mann–Whitney U, N = 106)** |  |  |  |
| Right PSV | U = 1195, Z = −0.730 | 0.465 | — |
| Left PSV | U = 1160.5, Z = −0.957 | 0.339 | — |
| **EDV at best PSV across BMI categories (Kruskal–Wallis, evaluable subset N = 74)** |  |  |  |
| Right EDV | H = 2.162, df = 2 | 0.339 | 0.352 (0.340–0.364) |
| Left EDV | H = 0.315, df = 2 | 0.854 | 0.855 (0.846–0.864) |
| **EDV at best PSV by arterial insufficiency status (Mann–Whitney U, N = 79; 69 vs 10)** |  |  |  |
| Right EDV | U = 285.5, Z = −0.877 | 0.388 | — |
| Left EDV | U = 256.5, Z = −1.305 | 0.196 | — |
| **EDV at best PSV by OSA status (Mann–Whitney U, N = 79; 67 vs 12)** |  |  |  |
| Right EDV | U = 324.5, Z = −1.059 | 0.295 | 0.292 (0.281–0.304) |
| Left EDV | U = 375, Z = −0.369 | 0.718 | 0.716 (0.705–0.728) |
| **EDV at best PSV by active stress (Mann–Whitney U, N = 79; 68 vs 11)** |  |  |  |
| Right EDV | U = 359.5, Z = −0.205 | 0.837 | 0.846 (0.836–0.855) |
| Left EDV | U = 321, Z = −0.751 | 0.453 | 0.458 (0.445–0.471) |
| **EDV at best PSV by psychiatric history (Mann–Whitney U, N = 79)** |  |  |  |
| Right EDV | U = 664, Z = −0.803 | 0.422 | — |
| Left EDV | U = 586.5, Z = −1.582 | 0.114 | — |
| **Spearman correlations: BMI vs hemodynamics** |  |  |  |
| BMI vs right PSV (N = 100) | ρ = −0.206 | **0.040** | — |
| BMI vs left PSV (N = 100) | ρ = −0.056 | 0.581 | — |
| BMI vs right EDV at best PSV, evaluable subset (N = 74) | ρ = 0.157 | 0.181 | — |
| BMI vs left EDV at best PSV, evaluable subset (N = 74) | ρ = 0.078 | 0.509 | — |
| BMI vs right EDV at best PSV, full cohort (N = 100) | ρ = 0.073 | 0.469 | — |
| BMI vs left EDV at best PSV, full cohort (N = 99) | ρ = 0.052 | 0.611 | — |

*PSV = peak systolic velocity; EDV = end-diastolic velocity; AI = arterial insufficiency; OSA = obstructive sleep apnea; BMI = body mass index. For each corpus cavernosum, the timepoint yielding the highest PSV was selected and the EDV at that same timepoint was used. EDV analyses are restricted to the 79 men with a final hardness score ≥ 5/10. Monte Carlo estimates are based on 10,000 sampled tables with 99% confidence intervals and were obtained for all comparisons involving the smallest groups; every asymptotic and Monte Carlo result was concordant. Bolded values indicate statistical significance.*
