# Supplementary material for: The impact of obesity and obstructive sleep apnea on vascular hemodynamics in men under 40: a 15-year retrospective cohort study
Source: Sex Med. 2026 Sep 14;14(6):qfag086. doi: 10.1093/sexmed/qfag086 (PMC13574288; doi:10.1093/sexmed/qfag086)
Supplement: Supplementary file 3 [file supplementary-table-s4-median-hemodynamic-values-by-exposure-group_qfag086.docx]

**Supplementary Table S4. Median Hemodynamic Values by Exposure Group**

| **Group** | **Right PSV, median (IQR)** | **Left PSV, median (IQR)** |
| --- | --- | --- |
| **BMI category (N = 100)** |  |  |
| Normal weight (n = 42) | 54.8 (37.2–79.8) | 48.7 (35.1–60.1) |
| Overweight (n = 42) | 45.7 (35.6–61.4) | 42.6 (38.8–68.3) |
| Obese (n = 16) | 41.9 (29.4–51.1) | 41.5 (28.1–59.7) |
| **OSA status (N = 106)** |  |  |
| No OSA (n = 93) | 48.6 (35.3–67.7) | 47.7 (36.2–62.9) |
| OSA (n = 13) | 40.7 (31.4–50.4) | 38.1 (28.7–50.2) |

| **Group** | **Right EDV at best PSV, median (IQR)** | **Left EDV at best PSV, median (IQR)** |
| --- | --- | --- |
| **Arterial insufficiency status (evaluable subset N = 79)** |  |  |
| Normal (n = 69) | 0.2 (−10.3 to 7.7) | 0.0 (−8.9 to 5.0) |
| Any AI (n = 10) | 0.6 (−3.0 to 5.2) | 2.4 (−1.0 to 6.6) |
| **OSA status (evaluable subset N = 79)** |  |  |
| No OSA (n = 67) | 0.0 (−10.2 to 7.6) | 0.0 (−7.9 to 5.0) |
| OSA (n = 12) | 4.0 (−5.0 to 8.8) | 1.2 (−7.7 to 5.8) |
| **BMI category (evaluable subset N = 74)** |  |  |
| Normal weight (n = 31) | 0.4 (−11.9 to 5.9) | 0.0 (−8.6 to 5.0) |
| Overweight (n = 30) | 0.0 (−7.9 to 8.2) | 0.3 (−7.6 to 6.0) |
| Obese (n = 13) | 4.1 (−4.4 to 9.8) | 2.4 (−7.4 to 6.3) |

*PSV = peak systolic velocity; EDV = end-diastolic velocity; AI = arterial insufficiency; OSA = obstructive sleep apnea. Values are median (25th–75th percentile), all in cm/s, and accompany the corresponding significance tests in Supplementary Table S3.*
