# Supplementary material for: The impact of obesity and obstructive sleep apnea on vascular hemodynamics in men under 40: a 15-year retrospective cohort study
Source: Sex Med. 2026 Sep 14;14(6):qfag086. doi: 10.1093/sexmed/qfag086 (PMC13574288; doi:10.1093/sexmed/qfag086)
Supplement: Supplementary file 4 [file supplementary-table-s5-association-between-traditional-risk-factors-labora_qfag086.docx]

**Supplementary Table S5. Association Between Traditional Risk Factors, Laboratory Markers, and Arterial Insufficiency**

| **Variable** | **Statistical test** | **p-value** | **Effect size** |
| --- | --- | --- | --- |
| **Traditional clinical diagnoses (N = 106)** |  |  |  |
| Hypertension × AI | Fisher's exact | 0.575 | — |
| Diabetes × AI | Fisher's exact | 0.711 | — |
| Dyslipidemia × AI | Fisher's exact | 0.309 | — |
| Smoking × AI | Fisher's exact | 0.854 | — |
| **Categorized laboratory markers** |  |  |  |
| Total cholesterol × AI (N = 49) | Fisher's exact | 0.830 | V = 0.075 |
| LDL cholesterol × AI (N = 48) | Fisher's exact | 0.445 | V = 0.154 |
| HDL cholesterol × AI (N = 49) | Fisher's exact | **0.035** | V = 0.478 |
| Triglycerides × AI (N = 49) | Fisher's exact | 0.085 | V = 0.338 |
| HbA1c × AI (N = 47) | Fisher's exact | 0.243 | — |
| **Continuous laboratory values across AI categories** |  |  |  |
| Total testosterone (N = 99) | Kruskal–Wallis | > 0.05 | — |
| Free testosterone (N = 91) | Kruskal–Wallis | > 0.05 | — |

**HDL category × arterial insufficiency cross-tabulation (N = 49)**

| **Arterial insufficiency** | **Normal HDL (n = 41)** | **Low HDL (n = 8)** |
| --- | --- | --- |
| Normal | 37 (90.2%) | 6 (75.0%) |
| Unilateral AI | 4 (9.8%) | 0 (0.0%) |
| Bilateral AI | 0 (0.0%) | 2 (25.0%) |

*AI = arterial insufficiency. Fisher's exact p = 0.035; Cramér's V = 0.478. The HDL association is limited by the small number of patients with available measurements and should be regarded as exploratory. Laboratory samples were drawn during routine care without protocolized timing or documented fasting status. Bolded values indicate statistical significance.*
