# Supplementary material for: The impact of obesity and obstructive sleep apnea on vascular hemodynamics in men under 40: a 15-year retrospective cohort study
Source: Sex Med. 2026 Sep 14;14(6):qfag086. doi: 10.1093/sexmed/qfag086 (PMC13574288; doi:10.1093/sexmed/qfag086)
Supplement: Supplementary file 5 [file supplementary-table-s6-age-trimix-volume-and-final-hardness-score-across-a_qfag086.docx]

**Supplementary Table S6. Age, Trimix Volume, and Final Hardness Score Across Arterial Insufficiency Categories (N = 106)**

| **Variable** | **Normal (n = 88)** | **Unilateral AI (n = 13)** | **Bilateral AI (n = 5)** | **H (df = 2)** | **p-value** |
| --- | --- | --- | --- | --- | --- |
| Age (years) | 30.8 (25.5–35.4) | 29.0 (25.8–34.1) | 32.4 (23.9–36.7) | 0.117 | 0.943 |
| Trimix volume (mL) | 0.05 (0.05–0.05) | 0.05 (0.05–0.10) | 0.10 (0.04–0.10) | 3.005 | 0.223 |
| Final hardness score (0–10) | 6.0 (5.0–8.0) | 5.0 (3.0–7.8) | 5.0 (3.3–6.5) | 1.108 | 0.575 |

*AI = arterial insufficiency. Values are median (25th–75th percentile); comparisons by Kruskal–Wallis test. The final hardness score is the post-redose value where a redose was administered. Trimix volume corresponds to papaverine 1.5 mg, phentolamine 0.05 mg, and alprostadil 0.5 mcg at the 0.05 mL median.*
