# Supplementary material for: The impact of obesity and obstructive sleep apnea on vascular hemodynamics in men under 40: a 15-year retrospective cohort study
Source: Sex Med. 2026 Sep 14;14(6):qfag086. doi: 10.1093/sexmed/qfag086 (PMC13574288; doi:10.1093/sexmed/qfag086)
Supplement: Supplementary file 6 [file supplementary-table-s7-sensitivity-analysis-excluding-patients-on-hormonal_qfag086.docx]

**Supplementary Table S7. Sensitivity Analysis Excluding Patients on Hormonal Therapy**

| **Analysis** | **Full cohort, Fisher's exact p** | **Excluding hormonal therapy, Fisher's exact p** |
| --- | --- | --- |
| AI × BMI category | 0.016 (N = 100) | **0.015 (N = 94)** |
| Venous leak × BMI category | 0.162 (N = 74) | 0.244 (N = 69) |

*AI = arterial insufficiency; BMI = body mass index. Seven patients receiving testosterone or clomiphene were excluded. In the arterial insufficiency analysis, 99 patients remained after exclusion, of whom 94 had BMI available; in the venous leak analysis, 73 of the evaluable patients remained, of whom 69 had BMI available. Venous leak was defined as end-diastolic velocity > 5 cm/s at the timepoint of best PSV and analyzed as a binary variable. The principal findings were unchanged. Bolded value indicates statistical significance.*
