# Supplementary material for: The impact of obesity and obstructive sleep apnea on vascular hemodynamics in men under 40: a 15-year retrospective cohort study
Source: Sex Med. 2026 Sep 14;14(6):qfag086. doi: 10.1093/sexmed/qfag086 (PMC13574288; doi:10.1093/sexmed/qfag086)
Supplement: Supplementary file 7 [file table-s2-association-between-psychological-factors-and-arter_qfag086.docx]

**Supplementary Table S2. Association Between Psychological Factors and Arterial Insufficiency (N = 106)**

| **Factor / category** | **Normal AI (n = 88)** | **Unilateral AI (n = 13)** | **Bilateral AI (n = 5)** | **p-value** | **Cramér's V** |
| --- | --- | --- | --- | --- | --- |
| **Active psychosocial stress** |  |  |  | 0.701 | 0.073 |
| No | 76 | 12 | 4 |  |  |
| Yes | 12 | 1 | 1 |  |  |
| **Any psychiatric history** |  |  |  | 0.179 | 0.190 |
| No | 52 | 11 | 4 |  |  |
| Yes | 36 | 2 | 1 |  |  |
| **Depression specifically** |  |  |  | 0.625 | 0.135 |
| No | 67 | 11 | 5 |  |  |
| Yes | 21 | 2 | 0 |  |  |

*AI = arterial insufficiency. All comparisons by Fisher's exact test.*
